# Correction: Essentially All Excess Fibroblast Cholesterol Moves from Plasma Membranes to Intracellular Compartments

**DOI:** 10.1371/journal.pone.0106160

**Published:** 2014-08-18

**Authors:** The PLOS ONE Staff

## Notice of Republication

This article was republished on July 25th, 2014, due to incorrect information being published in the Funding statement. The originally published, uncorrected article and the republished, corrected article are provided here for reference.

## Supporting Information

File S1 Originally published, uncorrected article.
(PDF)

File S2 Republished corrected article.
(PDF)
