# Supplementary material for: Correction: Essentially All Excess Fibroblast Cholesterol Moves from Plasma Membranes to Intracellular Compartments
Source: PLoS One. 2014 Aug 18;9(8):e106160. doi: 10.1371/journal.pone.0106160 (PMC4136908; doi:10.1371/journal.pone.0106160)
Supplement: File S1 — Originally published, uncorrected article. (PDF) [file pone.0106160.s001.pdf]

PLOS ONE

Essentially All Excess Fibroblast Cholesterol Moves from Plasma Membranes to Intracellular Compartments

Yvonne Lange[1]*, Jin Ye[1], Theodore L. Steck[2]

1 Department of Pathology, Rush University Medical Center, Chicago, Illinois, United States of America, **2** Department of Biochemistry and Molecular Biology, University of Chicago, Chicago, Illinois, United States of America

Abstract

It has been shown that modestly increasing plasma membrane cholesterol beyond its physiological set point greatly increases the endoplasmic reticulum and mitochondrial pools, thereby eliciting manifold feedback responses that return cell cholesterol to its resting state. The question arises whether this homeostatic mechanism reflects the targeting of cell surface cholesterol to specific intracellular sites or its general equilibration among the organelles. We now show that human fibroblast cholesterol can be increased as much as two-fold from 2-hydroxypropyl-β-cyclodextrin without changing the size of the cell surface pool. Rather, essentially all of the added cholesterol disperses rapidly among cytoplasmic membranes, increasing their overall cholesterol content by as much as five-fold. We conclude that the level of plasma membrane cholesterol is normally at capacity and that even small increments above this physiological set point redistribute essentially entirely to intracellular membranes, perhaps down their chemical activity gradients.

Citation: Lange Y, Ye J, Steck TL (2014) Essentially All Excess Fibroblast Cholesterol Moves from Plasma Membranes to Intracellular Compartments. PLoS ONE 9(7): e98482. doi:10.1371/journal.pone.0098482

Editor: David Holowka, Cornell University, United States of America

Received January 13, 2014; **Accepted** May 2, 2014; **Published** July 11, 2014

Copyright: © 2014 Lange et al. This is an open-access article distributed under the terms of the Creative Commons Attribution License, which permits unrestricted use, distribution, and reproduction in any medium, provided the original author and source are credited.

Funding: Funding was provided by the Commonwealth Scientific and Industrial Research Organisation (CSIRO) Capability Development Fund Ernst Frohlich Fellowship. HP was supported by a CSIRO Office of the Executive Chief Postdoctoral Award. BDB's time on this paper was supported by the Australia Governments' National Environmental Research Program, Marine Biodiversity Hub. The funders had no role in study design, data collection and analysis, decision to publish, or preparation of the manuscript.

Competing Interests: The authors have declared that no competing interests exist.

* Email: ylange@rush.edu

Introduction

Sterols are inhomogeneously distributed among the membranes of eukaryotic cells by unknown mechanisms [1–5]. PMs (plasma membranes) are characteristically sterol rich; in some animal cells, they contain ~80% of the total unesterified cell cholesterol at a CH/PL (cholesterol/phospholipid) mole ratio of 0.7–0.8 [6–8]. In contrast, the ER (endoplasmic reticulum) in some animal cells contains on the order of 1% of cell cholesterol at a CH/PL mole ratio of ~0.05 [9–11]. Cell cholesterol is not static but moves between organelles on a timescale of minutes [9,12–15]. A number of proteins in yeast and animal cells have been implicated in this intracellular flux, but it is not clear if any of them apportion sterol molecules to specific intracellular sites or pump sterols against their chemical activity gradients [13,16–21]. Rather, these proteins may facilitate the unenergized circulation of sterols among the cell membranes, so that the equilibrium sterol distribution is determined by its relative affinity for the diverse phospholipids in the various organelles.

The level of cholesterol in plasma membranes appears to be limited by the capacity of the high abundance, high affinity phospholipids with which it complexes [15,22]. Modest increments in plasma membrane cholesterol above this physiological set point cause the level of ER cholesterol to increase sharply by about an order of magnitude [9,23]. ER regulatory proteins then respond homeostatically to this signal so as to reduce the sterol excess [9–11,15]. A similar feedback mechanism is seen in mitochondria [14]. In that case, cholesterol that modestly exceeds the cell's physiological set point stimulates the synthesis of 27-hydroxycholesterol several-fold; this oxysterol then elicits a reduction in the level of cellular cholesterol through several feedback pathways [15]. Cell cholesterol homeostasis could thus be directed by the down-hill transfer of super-threshold cholesterol to regulatory sites in cytoplasmic membranes.

We have now tested a null hypothesis: that the large responses to excess plasma membrane cholesterol observed in the ER and mitochondria reflect a similar increase in the cholesterol in all of the intracellular membrane compartments, driven by its passive equilibration. Our data support this hypothesis: doubling the cholesterol in cultured human fibroblasts does not significantly increase their PM pool size; rather, essentially all of the extra sterol moves to cytoplasmic membranes.

Materials and Methods

Materials

Cholesterol was obtained from Steraloids and phospholipids from Avanti Polar Lipids. HPCD (ave. MW = 1396) and methyl-β-cyclodextrin (ave. MW = 1310) were purchased from Sigma-Aldrich. Cholesterol oxidase (EC 1.1.3.6; *Streptomyces* sp; reported activity of 43.9 U/mg protein) was obtained from EMD. The BCA protein assay kit was from Pierce.

Cell cholesterol compartmental analysis. Human fibroblasts were derived from the foreskins of circumcised infants at Rush University Medical Center by a procedure approved in writing by its Institutional Review Board. There are no records of these subjects. The cells were cultured in DME 10 (Dulbecco's modified Eagle's medium with 10% fetal bovine serum) to ~90%

confluence [24]. Cell cholesterol was modified as described in the legends to Figures 1 and 2 and ref. [8]. For cholesterol oxidase time courses, the cultures were rinsed and dissociated by a 1 min incubation at 37°C with 0.05% trypsin and 0.02% EDTA in PBS, pH 8.0. The cells were washed, fixed for 30 min on ice with 1% glutaraldehyde (to speed up the subsequent enzyme reaction), washed again and resuspended in PBS (pH 6.6) [8]. The cells were preincubated for 5 min at 37°C and oxidation time courses initiated at 37°C by the addition of 40 IU/ml of cholesterol oxidase. At intervals, aliquots were extracted for the analysis of the masses of the unesterified cholesterol and the cholest-4-en-3-one (cholestenone) oxidation product, the sum of which was essentially constant throughout each time course (SD of 4-7% in the 10 experiments shown in Figure 2). Data were expressed as % oxidation; *i.e.*, 100 x µg cholestenone/(µg cholesterol + µg cholestenone). The oxidation time courses were fit to a bi-exponential expression using SigmaPlot to obtain the oxidation rate constants for and the sizes of the rapidly- and slowly-oxidized pools [8]. The percent of cholesterol oxidation at the final time point, always 90–100% of the input, was taken as the value at infinite time.

Analytical procedures

Cholesterol and cholestenone were determined by HPLC [25]. Phospholipid P was determined by phosphomolybdate colorimetry on organic extracts [26,27]. Protein was determined using BCA with BSA as a standard. All assays were carried out at least in duplicate.

Results

Determination of the distribution of cellular cholesterol

Measuring the cholesterol content of individual organelles was not feasible. Instead, we measured the relative size of the pools of unesterified cholesterol in the plasma membrane and the intracellular compartments. We utilized the biphasic kinetics of cholesterol oxidase attack at the cell surface to estimate these two compartments [8]. The method employs glutaraldehyde fixation, to stimulate the oxidation reaction. We infer that the rapidly oxidized compartment is the cell surface cholesterol, while the slower compartment is the intracellular organelles, the cholesterol

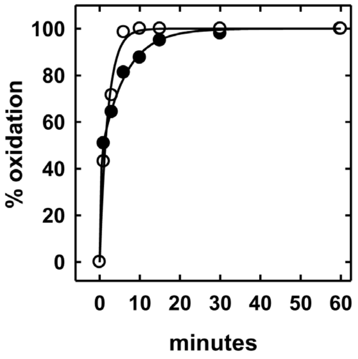

Figure 1. Representative time courses for the oxidation of intact fibroblast cholesterol. Replicate flasks were preincubated for 3 h at 37°C with DME containing 5% LPDS without (○) or with 1.3 mM HPCD complexed with 0.16 mM cholesterol (●). The control and enriched cells, containing 29 and 53 µg cholesterol/mg cell protein, respectively, were fixed, washed, incubated with cholesterol oxidase at 37°C and aliquots extracted at the designated intervals for analysis, as described in Methods and ref. [8].
doi:10.1371/journal.pone.0098482.g001

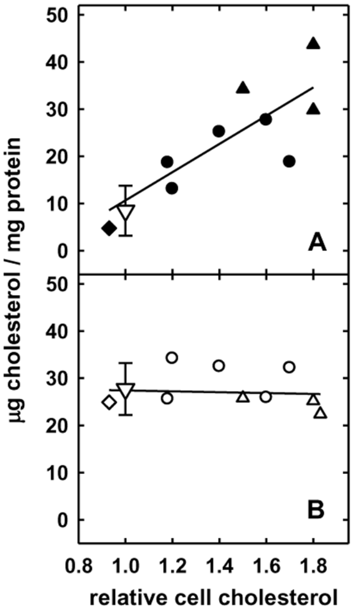

Figure 2. Influence of cell cholesterol on plasma membrane and intracellular cholesterol. To increase cell cholesterol, flasks were incubated for 8 min at 37°C with DME containing 5% LPDS plus 2.2 mM HPCD bearing 0.25 mM cholesterol (●, ○) or for 3 h with 1.3 mM HPCD bearing 0.08 mM cholesterol (▲, Δ). In addition, cell cholesterol was reduced by 7% by incubating a flask for 30 min at 37°C with DME containing 5% LPDS plus 4.3 mM HPCD (♦, ◇). The cells were fixed and the kinetics of oxidation analyzed as in Figure 1. Intracellular (Panel A) and plasma membrane cholesterol (Panel B) are plotted versus the relative total cell cholesterol in each of 10 experiments, and the plots were provided with linear least squares best fits. The means (∇) and standard deviation bars at the left of each panel represent the matched untreated control cells for these experiments.
doi:10.1371/journal.pone.0098482.g002

in which circulates through the cell surface compartment during the incubation with the enzyme. Interpretation of the data also assumes the rapid flip-flop of cholesterol across the plasma membrane and the mobility of the entire sterol pool within the fixed cells [8,28]. The method was validated by an independent technique in a previous study using Niemann Pick C cells that bear large, slowly-circulating endolysosomal cholesterol pools [8].

Applying this approach to normal human skin fibroblasts was more challenging because of the small size of the intracellular cholesterol pool and its more rapid circulation. Nevertheless, the method worked well, as illustrated in Figure 1. While first-order least-squares fits to oxidation time courses were not satisfactory, bi-exponential fits always gave values of R^2 of >0.99. The fast (plasma membrane) component was oxidized with half-times of ~1 min in control cells and ~0.5 min in enriched cells, while the slower (intracellular) pool was oxidized as it circulated to the cell surface with a half-time of ~7 min in control cells and ~3.5 min in the enriched cells. Since the cells were fixed and the circulation rapid, we infer that cholesterol was transferred by a non-vesicular pathway [12,19]. On average, control cells contained 35.0±3.9 (SD, n = 10) µg total cholesterol/mg protein. Of this, the PM fraction constituted 76% [*i.e.*, 26.6±5.3 (SD, n = 9)] µg cholesterol/mg protein); the remaining 24% [*i.e.*, 8.4±4.7 (SD, n = 9) µg cholesterol/mg protein] was ascribed to intracellular compartments. These results, including the observation that intracellular cholesterol is fully mobile in glutaraldehyde-fixed cells, are in accord with earlier findings [8]. The surprisingly rapid transfer of

cholesterol among the organelles within fixed cells could be mediated by membrane contact sites that circumvent the diffusion of cytoplasmic carrier proteins [18,19].

Dependence of cholesterol distribution on total cell cholesterol

In various experiments, we increased the cell cholesterol up to two-fold (from ~35 to ~70 μg cholesterol/mg protein) by incubation with HPCD bearing different loads of cholesterol. Incubation for 8 min with a high level of cholesterol enriched the cells about as much as a 3 h incubation with a moderate level of cholesterol, in keeping with other evidence for the rapid equilibration of sterols between β-cyclodextrin donors and PMs at 37°C [28,29]. Increasing the cell cholesterol did not appreciably affect the subsequent growth of the cells or visibly perturb them. (The maximal load of cholesterol per phospholipid introduced into the PMs here was less than that achieved with red blood cells, also without ill effect [30].) In control experiments (not shown), a doubling of the cell cholesterol was reversed by overnight incubation in growth medium (with a half-time of ~5 h) and by incubation with methyl-β-cyclodextrin for 90 min in DME containing 5% LPDS. The cells grew normally after this reversal.

Surprisingly, the uptake of exogenous cholesterol did not increase the size of the PM pool (Figure 2). That is, the PM of the enriched cells remained essentially unchanged, containing 27.0±4.4 (SD, n = 9) μg cholesterol/mg protein after the various loadings, compared to 26.6 μg cholesterol/mg protein in the control cells. In contrast, the intracellular pool took up virtually all of the cholesterol increment, increasing as much as 5-fold from control values of 8.4 to 43.7 μg cholesterol/mg cell protein. This change in the distribution of cell cholesterol was not caused by experimental factors such as the chilling or fixing of the cells or exposure to HPCD, since it did not occur in unenriched cells or those treated with HPCD alone but, rather, was directly proportional to the cholesterol load. Furthermore, glutaraldehyde fixation immobilizes the cytoplasm so as to render cells impermeable to proteins [31] as we demonstrated in a previous study [8,31].

Time course of cholesterol uptake by the ER

To test whether excess cholesterol can move rapidly from the PM to the cell interior in these cells, we followed the response of the ER to a jump in PM cholesterol. The approach involved applying a large pulse of exogenous cholesterol for 6 minutes at 37°C, homogenizing the cells and marking the cholesterol residing in the fragments of ER bearing acyl-CoA:cholesterol acyltransferase by allowing its complete conversion to the radioactive ester form by incubation with [^{14}C]oleoyl-CoA [9]. As shown in Figure 3, the size of the unesterified ER pool was found to increase more than 15-fold during the brief interval that followed the jump in plasma membrane cholesterol. These data support the implication of Figure 2 that increasing cell cholesterol greatly expands cytoplasmic membrane pools. (Previous studies have shown similar large excursions in ER cholesterol with cell loading, but the kinetics were not analyzed in those experiments [9,10,23].)

Control experiments

We considered the possibility that the large, slowly oxidized pool of excess cholesterol in Figures 1 and 2 was not intracellular but, rather, a sluggish PM compartment. We tested this hypothesis by performing the cholesterol oxidase reaction on homogenates of cholesterol-loaded cells. In that case, all of the cholesterol became completely oxidized with a single rate constant at least as fast as

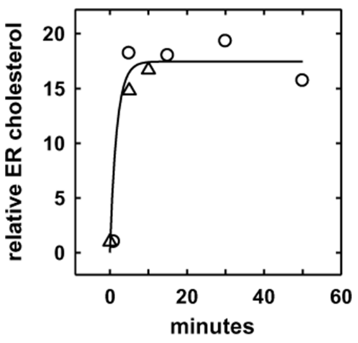

Figure 3. Response of endoplasmic reticulum cholesterol to a jump in PM cholesterol. Replicate flasks were preincubated for 6 min at 37°C in PBS with or without 3 mM HPCD bearing 0.33 mM cholesterol. The cells were then quickly rinsed with PBS at room temperature, overlaid with PBS and chased at 37°C. At the designated times of chase, the cells in each flask were harvested and homogenized and total cell cholesterol, cell protein, and unesterified ER cholesterol quantified as described [9]. Ordinate values indicate the ratio of enriched to control cell ER cholesterol. The symbols designate two separate experiments. The zero time points were unenriched controls. Control cells had 27.0 and 26.7 μg cholesterol/mg protein; the multiple enriched flasks in the two time courses had averages of 42.6±4.2 SD (n = 3) and 48.5±2.2 SD (n = 4) μg cholesterol/mg protein.
doi:10.1371/journal.pone.0098482.g003

that for the rapid compartment in the intact cells (not shown). Further evidence on this point comes from a previous study showing that enrichment of PM cholesterol greatly increased its susceptibility to cholesterol oxidase [32]. This makes it implausible that the slow cholesterol pool in enriched cells was a poorly-reactive PM compartment. We also tested whether the large intracellular pool inferred from Figure 2 manifested cholesterol binding by soluble cytoplasmic proteins [12,13,19]. We found, however, that all of the cholesterol detectable in homogenates prepared from both control and enriched cells was pelleted by centrifugation at 100,000 *g* for 2 h; hence, entirely particulate. We also observed (not shown) that enriched cells labeled with BODIPY-cholesterol had a normal morphology and distribution of membrane fluorescence; in particular, there was no precipitation of the label or conspicuous concentration in particular organelles such as lipid droplets or the endosomal recycling compartment, ERC [13,33].

The disposition of the intracellular cholesterol was further analyzed by centrifuging homogenates to equilibrium on sucrose density gradients; a representative experiment is shown in Figure 4. The sterol in both the control and loaded cells was broadly distributed in the gradient with a peak at ~33% sucrose that coincides with the plasma membrane markers previously established for this system [7,34]. Panel A shows that the cholesterol increment in the treated cells was more broadly distributed through the gradient than the control, suggesting that it was not confined to either the plasma membrane or a specific subcellular locus. (The homogenate of the enriched cells contained 67% more cholesterol per protein than the control; similarly, the cholesterol recovered from the gradient fractions of the enriched cells was 59% greater than that for the control cells; this indicates that there was no significant loss of cholesterol in the gradient.)

The peak CH/PL mole ratio in the control cells in Figure 4B was 0.74, characteristic of purified plasma membranes [6,7]. The CH/PL mole ratio for the enriched cells was elevated throughout the gradient; while it was only modestly increased in the PM region, it was 2.4-fold higher than the control in the buoyant fractions. These results are in keeping with the hypothesis that the

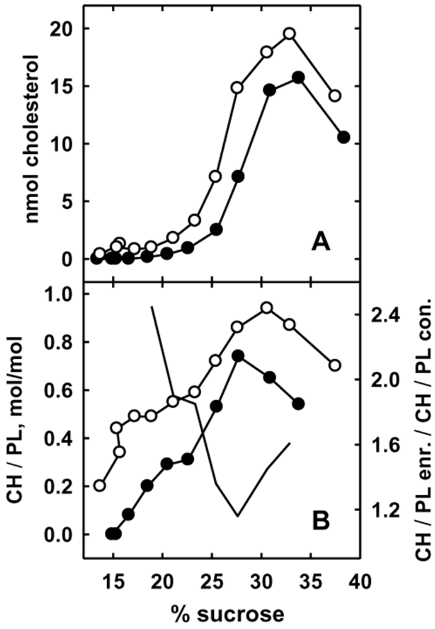

Figure 4. Gradient distribution of cholesterol in homogenates of control versus cholesterol-enriched fibroblasts. Replicate flasks of fibroblasts were incubated at 37°C for 3.5 h in LPDS in the absence (●) or presence (○) of 1.2 mM HPCD bearing 0.12 mM cholesterol. The control and enriched cells contained 42 and 70 μg cholesterol/mg protein, respectively. The cells were washed, dissociated, homogenized and equivalent amounts of homogenate (measured as protein) spun to equilibrium on sucrose gradients [53]. The gradient fractions were extracted and analyzed for cholesterol (CH) and phospholipid (PL). Panel A, nmol cholesterol/fraction. Panel B, cholesterol/phospholipid, mole/mole, per fraction (left axis) and the ratios of the mole ratios in enriched versus control in fractions bearing significant amounts of material (right axis, no symbols). Abscissa, sucrose concentration as weight/volume. A representative experiment.
doi:10.1371/journal.pone.0098482.g004

exogenous cholesterol neither remained in the plasma membranes nor precipitated but, rather, became broadly distributed among the intracellular organelles.

Modeling the fate of cholesterol in enriched cells. We used a simple simulation to aid the interpretation of the findings presented in Figure 2. We assumed, as justified below, that cholesterol forms 1:1 complexes with phospholipids in the PM (o) and 1:3 complexes with those in the intracellular membranes (i). Then,

$$CH_o + PL_o \longleftrightarrow (CH-PL)_o \tag{1}$$

$$K_1 = [CH-PL]_o/([CH_o][PL_o]) \tag{2}$$

$$CH_i + 3PL_i \longleftrightarrow (CH-PL_3)_i \tag{3}$$

$$K_2 = [CH-PL_3]_i/([CH_i][PL_i]^3) \tag{4}$$

We also assumed that uncomplexed cholesterol equilibrates between the outer and inner compartments:

$$CH_o \longleftrightarrow CH_i \tag{5}$$

This equilibrium distribution can depend in a complex and unknown fashion on the abundance and characteristics of the various bilayer phospholipids as well as on the amounts of cholesterol present [27]. We therefore defaulted to a simple partition coefficient, K_3:

$$K_3 = ([CH_o]/[PL_o])/([CH_i]/[PL_i]) \tag{6}$$

Figure 5 presents a simulation with $K_1 = 1{,}000$, $K_2 = 200$ and $K_3 = 0.1$. (Concentrations are in mole fractions.) The K_1 and K_2 values as well as the CH/PL stoichiometries employed were in line with literature values for cholesterol-phospholipid interactions [27]. For illustrative purposes, we let CH/PL = 0.37 for the whole unmodified fibroblasts and CH/PL = 0.69 for their PM (These values are marked by the cross in Figure 5). We also assumed that the PM contained 80–90% of the resting cell cholesterol and half of its phospholipid; see Results and refs. [7,8]. In this simulation, PM cholesterol begins to plateau near its physiological resting value just as in Figure 2, although the latter did so much more sharply. In Figure 5, as in Figure 2, the PM retained very little of the excess cholesterol, essentially all of it becoming intracellular. That is, nearly all of the PM cholesterol (◇) was in complexes (●) and not free (○). In addition, this simulation shows that there is very little uncomplexed intracellular cholesterol in unmodified cells (*i.e.*, in the region below CH/PL = 0.37) and that surplus cholesterol beyond the physiologic set point (cross) goes mostly to the uncomplexed intracellular pool. This prediction is in line with the demonstration of highly thresholded homeostatic responses to super-threshold cholesterol by cytoplasmic effectors—namely, cholesterol esterification in the ER and cholesterol hydroxylation in the mitochondria [9,14,15]. Finally, the CH/PL in the

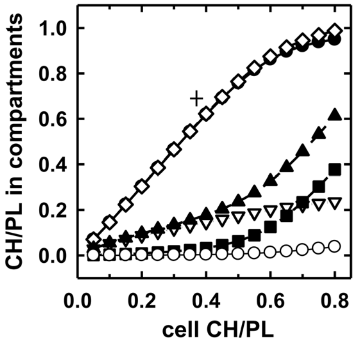

Figure 5. Simulation of the distribution of complexed and uncomplexed cholesterol in plasma membranes and intracellular membranes as a function of total cell cholesterol. CH/PL denotes moles cholesterol/mole phospholipid. Total plasma membrane cholesterol (◇); plasma membrane cholesterol complexes (●); total intracellular cholesterol (▲); uncomplexed intracellular cholesterol (■); complexed intracellular cholesterol (▽); uncomplexed plasma membrane cholesterol (○). The cross designates typical literature values for the CH/PL of unmodified intact fibroblasts and for the CH/PL of their plasma membranes. See the text for a discussion of the values employed.
doi:10.1371/journal.pone.0098482.g005

simulated intracellular compartment (▲) ultimately approached that in the PM (◇), as in Figure 2.

For the simulation to be concordant with the data, we had to assign a partition coefficient for the uncomplexed cholesterol (K_3) that strongly favored the cytoplasmic compartment. This would be the case if the "free" cholesterol in excess of the stoichiometric equivalence point was less soluble in the continuum of PM CH-PL complexes than in the cytoplasmic membranes. The literature suggests that the PM phospholipids would be significantly more avid for cholesterol than those in the intracellular membranes below the stoichiometric equivalence point for their complexation (*i.e.*, $K_1 > K_2$) [27]. The unexpected implication of Figures 2 and 5 is that the opposite relationship holds for the partition of the uncomplexed excess sterol (*i.e.*, $K_3 < 1$). This strong prediction requires testing; however, we currently lack detailed information concerning the abundance of the multitude of phospholipid species in the various organelles as well as their sterol stoichiometries and affinities both below and above their equivalence points. If the affinity of the cytoplasmic membranes for uncomplexed cholesterol is found not to be significantly higher than that of the plasma membrane, a different explanation must be sought for the results presented herein and in studies of the effect of cell cholesterol levels on its regulation [15].

Discussion

This study was prompted by evidence that the cholesterol pools in ER and mitochondria increase by about an order of magnitude when cell cholesterol is raised modestly [9,15,23]. Figure 2 affirms the null hypothesis that the bulk of cholesterol in excess of the physiological set point of the PM moves to the intracellular organelles at the expense of the cell surface compartment. That is, essentially all of the extra cholesterol load moved quantitatively from the PM to intracellular compartments, increasing their sterol content several-fold. (Some of the excess cholesterol must remain in the PM, however, since small increments greatly increase the susceptibility of the cell surface to cholesterol oxidase attack and to β-cyclodextrin extraction [32].)

Figure 3 shows that the transfer of PM cholesterol to the ER occurs on a time scale of a few minutes. Similar rapid kinetics for the decrease of ER cholesterol were observed when cholesterol was extracted from intact cells with HPCD [9]. These findings are consistent with a variety of other studies suggesting a brisk circulation of cholesterol among cell membranes [9,13,14,15]. The extra intracellular cholesterol was not dissolved in the cytosol or located in precipitates or in a single locus, but was distributed widely, particularly in buoyant membranes (Fig. 4). It is unlikely that the excess cholesterol was brought into the cell through a vesicular pathway, since the rate of uptake of cholesterol from 6 or 8-min pulses was orders of magnitude greater than endocytosis would predict [35]. Rather, the added cholesterol might move down its chemical activity gradient between the PM and the cell interior by any of several transport pathways [13,16–21].

The state of the surfeit of cholesterol in the cytoplasmic membranes can be viewed in two ways. One posits a phase separation or crystallization of excess sterol molecules [2,36,37]. Consistent with this premise, the unsaturated phospholipids enriched in the cytoplasmic membranes are known to have a low solubility limit for cholesterol compared to the saturated phosphorylcholine species characteristic of PMs [37,38]. This mechanism might also account for the sharp increase in cholesterol accessibility to probes such as cholesterol oxidase and β-cyclodextrins that parallels the solubility limits reported for various phospholipid species [27,37–40]. In fact, cholesterol free of association with phospholipids shows enhanced accessibility to cholesterol oxidase and β-cyclodextrin, at least in condensed monolayer films [41,42].

Arguing against the crystallization hypothesis is the report that more than a day was required for crystals to form from large loads of intracellular cholesterol [43]. It also seems unlikely that cholesterol crystals would serve as a homeostatic signal to regulatory proteins or be a substrate for homeostatic enzymes in the ER and mitochondria. Rather, crystallization should dissipate the elevated chemical potential that appears to drive the association of cholesterol with and consequent stimulation of these proteins; see, for example, refs. [9,11]. Furthermore, two recent molecular dynamics simulations found that excess cholesterol was not crystalline (albeit, using a brief time window) but rather dispersed in the bilayer with a high chemical activity [40,44].

An alternative view is that cholesterol is not simply an indifferent bilayer solute but rather forms (presumably weak and dynamic) complexes of varied stoichiometries and affinities with different membrane phospholipids. While such complexes have not been demonstrated directly, there is considerable evidence for specific chemical associations between sterols and phospholipids, as illustrated in ref. [45]. Cholesterol exceeding stoichiometric equivalence with the phospholipids would remain dissolved and dispersed in the bilayer of complexes in a metastable state [15,22,27,46]. Being less constrained by association with the phospholipids, super-threshold cholesterol molecules would have an elevated chemical activity [40,44]. This would promote their transfer to sites with a lower free energy; in this case, driving the sterol from over-loaded PMs to under-saturated intracellular compartments.

Supporting this hypothesis is diverse evidence for a sharp increase in the accessibility or activity of cholesterol exceeding a characteristic mole ratio with various bilayer phospholipids [15]. Thresholds are seen in diverse contexts: the susceptibility to cholesterol oxidase of cholesterol in PMs and/or phospholipid vesicles; its extraction by β-cyclodextrin; its binding to perfringolysin O toxin; its 27-hydroxylation in mitochondria; its rise in abundance in the ER and the consequent rise in the activation of ER proteins that it regulates [10,11,22,23,27,47,48]. In addition, numerous intercalating amphipaths appear to compete stoichiometrically with and displace cholesterol from phospholipids, providing independent evidence for phospholipid complexation and the enhanced activity and/or accessibility of uncomplexed cholesterol [49,50].

The two aforementioned hypotheses might actually depict different features of the same process. Consider that cholesterol in excess of the capacity of the phospholipids would remain soluble and dispersed in the bilayers of those complexes. [The highest level of exogenous cholesterol introduced in these experiments would have led to an overall cellular CH/PL of ≤0.8 (a cholesterol mole fraction of ≤0.44) in both the plasma membranes and cytoplasmic organelles—not an unprecedented bilayer load.] The high chemical activity of the super-threshold fraction could drive its redistribution to other membranes and ligands. In the absence of such alternative opportunities, excess sterol would eventually reach the limit of its solubility in the bilayer and dissipate its high chemical activity through crystallization.

Our data support the view that increments in cell cholesterol will preferentially associate with the high capacity, high-affinity phospholipids in the PM at the expense of the phospholipids in the cytoplasmic membranes with their lower affinity and capacity. Cholesterol rising beyond the stoichiometric equivalence point of the PM phospholipids would be allocated wholesale to intracel-

lular membranes as in Figures 2 and 5. The consequent increment in ER and mitochondrial cholesterol would then associate with the regulatory proteins therein, eliciting homeostatic responses that limit further cholesterol accumulation [15]. Excess cellular cholesterol is toxic to cells, perhaps because of the formation of crystals and/or through ER stress [51,52]. By holding cholesterol levels at or below the saturation point for the phospholipids in the cell surface and intracellular bilayers, this feedback system can serve to protect cells from injury.

Acknowledgments

We thank Ali Tabei (University of Chicago) for his help with the simulations. We are also grateful to Diana Bach and Ellen Wachtel (Weizmann Institute, Rehovot, Israel) for calling our attention to the issue of membrane cholesterol crystal formation.

Author Contributions

Conceived and designed the experiments: YL TLS. Performed the experiments: YL JY. Analyzed the data: YL TLS. Contributed reagents/materials/analysis tools: YL. Wrote the paper: YL TLS.

References

1. Zinser E, Paltauf F, Daum G (1993) Sterol composition of yeast organelle membranes and subcellular distribution of enzymes involved in sterol metabolism. J Bacteriol 175: 2853–2858.
2. van Meer G, Voelker DR, Feigenson GW (2008) Membrane lipids: where they are and how they behave. Nat Rev Mol Cell Biol 9: 112–124.
3. Maxfield FR, van Meer G (2010) Cholesterol, the central lipid of mammalian cells. Current Opinion in Cell Biology 22: 422–429.
4. Goedeke L, Fernández-Hernando C (2012) Regulation of cholesterol homeostasis. Cellular and molecular life sciences: CMLS 69: 915–930.
5. Brown AJ (2012) Cholesterol versus other sterols: How do they compare as physiological regulators of cholesterol homeostasis? European Journal of Lipid Science and Technology 114: 617–623.
6. Steck TL, Wallach D (1970) The isolation of plasma membranes. Methods in Cancer Research 5: 93–153.
7. Lange Y, Swaisgood MH, Ramos BV, Steck TL (1989) Plasma membranes contain half the phospholipid and 90% of the cholesterol and sphingomyelin in cultured human fibroblasts. J Biol Chem 264: 3786–3793.
8. Lange Y, Ye J, Steck TL (2012) Activation mobilizes the cholesterol in the late endosomes-lysosomes of niemann pick type C cells. PLoS One 7: e30051.
9. Lange Y, Ye J, Rigney M, Steck TL (1999) Regulation of endoplasmic reticulum cholesterol by plasma membrane cholesterol. J Lipid Res 40: 2264–2270.
10. Radhakrishnan A, Goldstein JL, McDonald JG, Brown MS (2008) Switch-like Control of SREBP-2 Transport Triggered by Small Changes in ER Cholesterol: A Delicate Balance. Cell Metabolism 8: 512–521.
11. Sokolov A, Radhakrishnan A (2010) Accessibility of Cholesterol in Endoplasmic Reticulum Membranes and Activation of SREBP-2 Switch Abruptly at a Common Cholesterol Threshold. Journal of Biological Chemistry 285: 29480–29490.
12. Prinz WA (2007) Non-vesicular sterol transport in cells. Prog Lipid Res 46: 297–314.
13. Mesmin B, Maxfield FR (2009) Intracellular sterol dynamics. Biochimica et Biophysica Acta (BBA) - Molecular and Cell Biology of Lipids 1791: 636–645.
14. Lange Y, Steck TL, Ye J, Lanier MH, Molugu V, et al. (2009) Regulation of fibroblast mitochondrial 27-hydroxycholesterol production by active plasma membrane cholesterol. J Lipid Res 50: 1881–1888.
15. Steck TL, Lange Y (2010) Cell cholesterol homeostasis: Mediation by active cholesterol. Trends in Cell Biology 20: 680–687.
16. Soccio RE, Breslow JL (2004) Intracellular cholesterol transport. Arterioscler Thromb Vasc Biol 24: 1150–1160.
17. Ikonen E (2008) Cellular cholesterol trafficking and compartmentalization. Nat Rev Mol Cell Biol 9: 125–138.
18. Raychaudhuri S, Prinz WA (2010) The diverse functions of oxysterol-binding proteins. Annu Rev Cell Dev Biol 26: 157–177.
19. Lev S (2010) Non-vesicular lipid transport by lipid-transfer proteins and beyond. Nature Reviews Molecular Cell Biology 11: 739–750.
20. Georgiev AG, Sullivan DP, Kersting MC, Dittman JS, Beh CT, et al. (2011) Osh Proteins Regulate Membrane Sterol Organization but Are Not Required for Sterol Movement Between the ER and PM. Traffic 12: 1341–1355.
21. Wustner D (2012) Following Intracellular Cholesterol Transport by Linear and Non-Linear Optical Microscopy of Intrinsically Fluorescent Sterols. Current Pharmaceutical Biotechnology 13: 303–318.
22. Lange Y, Steck TL (2008) Cholesterol homeostasis and the escape tendency (activity) of plasma membrane cholesterol. Prog Lipid Res 47: 319–332.
23. Lange Y, Steck TL, Ye J, Lanier MH, Molugu V, et al. (2009) Regulation of fibroblast mitochondrial 27-hydroxycholesterol production by active plasma membrane cholesterol. J Lipid Res 50: 1881–1888.
24. Echevarria F, Norton RA, Nes WD, Lange Y (1990) Zymosterol is located in the plasma membrane of cultured human fibroblasts. J Biol Chem 265: 8484–8489.
25. Lange Y, Echevarria F, Steck TL (1991) Movement of zymosterol, a precursor of cholesterol, among three membranes in human fibroblasts. J Biol Chem 266: 21439–21443.
26. Chen PS, Toribara TY, Warner H (1956) Microdetermination of Phosphorus. Analytical Chemistry 28: 1756–1758.
27. Lange Y, Tabei SMA, Ye J, Steck TL (2013) Stability and Stoichiometry of Bilayer Phospholipid-Cholesterol Complexes: Relationship to Cellular Sterol Distribution and Homeostasis. Biochemistry 52: 6950–6959.
28. Steck TL, Ye J, Lange Y (2002) Probing red cell membrane cholesterol movement with cyclodextrin. Biophys J 83: 2118–2125.
29. Wustner D, Herrmann A, Hao M, Maxfield FR (2002) Rapid nonvesicular transport of sterol between the plasma membrane domains of polarized hepatic cells. J Biol Chem 277: 30325–30336.
30. Cooper RA (1978) Influence of increased membrane cholesterol on membrane fluidity and cell function in human red blood cells. J Supramol Struct 8: 413–430.
31. Pitchiaya S, Krishnan V, Custer TC, Walter NG (2013) Dissecting non-coding RNA mechanisms in cellulo by Single-molecule High-Resolution Localization and Counting. Methods 63: 188–199.
32. Lange Y, Ye J, Steck TL (2004) How cholesterol homeostasis is regulated by plasma membrane cholesterol in excess of phospholipids. Proc Natl Acad Sci U S A 101: 11664–11667.
33. Hao M, Lin SX, Karylowski OJ, Wustner D, McGraw TE, et al. (2002) Vesicular and non-vesicular sterol transport in living cells. The endocytic recycling compartment is a major sterol storage organelle. J Biol Chem 277: 609–617.
34. Lange Y, Ye J, Steck TL (1998) Circulation of cholesterol between lysosomes and the plasma membrane. J Biol Chem 273: 18915–18922.
35. Murphy RF, Powers S, Cantor CR, Pollack R (1984) Reduced Insulin Endocytosis in Serum-Transformed Fibroblasts Demonstrated by Flow-Cytometry. Cytometry 5: 275–280.
36. Huang J, Buboltz JT, Feigenson GW (1999) Maximum solubility of cholesterol in phosphatidylcholine and phosphatidylethanolamine bilayers. Biochim Biophys Acta 1417: 89–100.
37. Bach D, Wachtel E (2003) Phospholipid/cholesterol model membranes: formation of cholesterol crystallites. Biochimica et Biophysica Acta (BBA) - Biomembranes 1610: 187–197.
38. Brzustowicz MR, Cherezov V, Caffrey M, Stillwell W, Wassall SR (2002) Molecular organization of cholesterol, in polyunsaturated membranes: Microdomain formation. Biophysical Journal 82: 285–298.
39. Ziblat R, Leiserowitz L, Addadi L (2010) Crystalline Domain Structure and Cholesterol Crystal Nucleation in Single Hydrated DPPC:Cholesterol:POPC Bilayers. Journal of the American Chemical Society 132: 9920–9927.
40. Lopez CA, de Vries AH, Marrink SJ (2013) Computational microscopy of cyclodextrin mediated cholesterol extraction from lipid model membranes. Scientific Reports 3: 1–6.
41. Slotte JP (1992) Enzyme-Catalyzed Oxidation of Cholesterol in Pure Monolayers at the Air-Water-Interface. Biochimica Et Biophysica Acta 1123: 326–333.
42. Ohvo H, Slotte JP (1996) Cyclodextrin-mediated removal of sterols from monolayers: Effects of sterol structure and phospholipids on desorption rate. Biochemistry 35: 8018–8024.
43. Tangirala RK, Jerome WG, Jones NL, Small DM, Johnson WJ, et al. (1994) Formation of cholesterol monohydrate crystals in macrophage-derived foam cells. Journal of Lipid Research 35: 93–104.
44. Olsen BN, Bielska AA, Lee T, Daily MD, Covey DF, et al. (2013) The structural basis of cholesterol accessibility in membranes. Biophys J 105: 1838–1847.
45. Aittoniemi J, Niemela PS, Hyvonen MT, Karttunen M, Vattulainen I (2007) Insight into the putative specific interactions between cholesterol, sphingomyelin, and palmitoyl-oleoyl phosphatidylcholine. Biophysical Journal 92: 1125–1137.
46. McConnell HM, Radhakrishnan A (2003) Condensed complexes of cholesterol and phospholipids. Biochimica Et Biophysica Acta-Biomembranes 1610: 159–173.
47. Ohno-Iwashita Y, Iwamoto M, Ando S, Iwashita S (1992) Effect of lipidic factors on membrane cholesterol topology—mode of binding of theta-toxin to cholesterol in liposomes. Biochim Biophys Acta 1109: 81–90.
48. Johnson BB, Moe PC, Wang D, Rossi K, Trigatti BL, et al. (2012) Modifications in Perfringolysin O Domain 4 Alter the Cholesterol Concentration Threshold Required for Binding. Biochemistry 51: 3373–3382.
49. Lange Y, Ye J, Duban ME, Steck TL (2009) Activation of membrane cholesterol by 63 amphipaths. Biochemistry 48: 8505–8515.
50. Olsen BN, Schlesinger PH, Ory DS, Baker NA (2011) 25-hydroxycholesterol increases the availability of cholesterol in phospholipid membranes. Biophys J 100: 948–956.

51. Kellner-Weibel G, Yancey PG, Jerome WG, Walser T, Mason RP, et al. (1999) Crystallization of free cholesterol in model macrophage foam cells. Arteriosclerosis Thrombosis and Vascular Biology 19: 1891–1898.
52. Feng B, Yao PM, Li Y, Devlin CM, Zhang D, et al. (2003) The endoplasmic reticulum is the site of cholesterol-induced cytotoxicity in macrophages. Nat Cell Biol 5: 781–792.
53. Lange Y, Ye J, Rigney M, Steck TL (2000) Cholesterol movement in Niemann-Pick type C cells and in cells treated with amphiphiles. J Biol Chem 275: 17468–17475.
